# Understanding dengue transmission by using participatory research and community-focused strategies for prevention and control in Bangladesh

**DOI:** 10.1186/1471-2334-12-S1-P92

**Published:** 2012-05-04

**Authors:** Parnali Dhar Chowdhury, Emdad Haque

**Affiliations:** 1 Natural Resources Institute, University of Manitoba, Winnipeg, Canada

## Background

Globally, dengue has become one of the most alarming infectious diseases and its resurgence reflects the failure of traditional reductionistic disciplinary approach. Because neither an effective vaccine nor an effective vector control program is available for dengue prevention and control, a Community-Based Approach (CBA) to vector control and individual behavior change has been implemented in many countries. However, by and large, the CBA has failed as it ignored local community members’ basic needs and perspectives in these processes. By challenging reductionist notions, this research will delineate the mental maps of local urban residents of the City of Dhaka, Bangladesh concerning dengue transmission and methods of dengue prevention and control.

## Methods

This study involved focus group discussion in 3 wards of Dhaka City Corporation, semi-structured interview of 30 stakeholders representatives; 900 ward/community members (300 from each ward); 18 policy- and/or decision makers (national and local institutions) and community members’ mental map construction of 24 ward representatives (supplemented by 300 ward members).

## Results

This study revealed the lack of intersectoral coordination between local and national institutions dealing with disease and household sanitation, and highlights the difficulties in avoiding dengue vectors in urban areas with irregular water supply, poor sanitation services, and finally the location of large and small construction zones all over the city.

## Conclusion

The conclusion emphasizes the importance of the knowledge about the daily problems faced by the community members and partnership needed in all sectors to address water supply problem and disease surveillance systems.

